# A Delphi-Based Exploratory Estimation of the Economic Impact of Coccidiosis in Turkish Broiler Production

**DOI:** 10.3390/ani16071096

**Published:** 2026-04-02

**Authors:** Seyfettin Tuncel, Pınar Demir Ayvazoğlu, Yasin Parlatır

**Affiliations:** 1 Department of Animal Health Economics and Management, Faculty of Veterinary Medicine, Kırıkkale University, Yahşihan 71450, Kırıkkale, Türkiye; pinardemir80@hotmail.com; 2 Department of Internal Medicine, Kırıkkale University, Yahşihan 71450, Kırıkkale, Türkiye; yasinparlatir@kku.edu.tr

**Keywords:** broiler, coccidiosis, delphi method, economic analysis, feed conversion ratio

## Abstract

This study provides a model-based exploratory assessment of the economic impact attributable to coccidiosis within the Turkish broiler sector. Primary data were obtained from 117 commercial enterprises (total capacity: 1,666,000 broilers) across the Mediterranean, Marmara, and Central Anatolia regions during the 2024 production cycle. Epidemiological analysis estimated a disease prevalence of 13% within the sampled population. The economic impact was evaluated using a stochastic modeling framework informed by Monte Carlo simulation and integrated with the Delphi method. Total national financial losses in Türkiye were estimated to reach a mean of $15.1 million in the most likely scenario (with a probabilistic range extending from $3.3 million under optimistic assumptions to $46.3 million under pessimistic conditions). The model-estimated average financial burden per animal was approximately $0.41 (representing an estimated $2.06 per clinically infected bird). Notably, projections suggest that roughly 62% of this economic impact could be attributed to subclinical factors, characterized by a modeled 5% (150 g) reduction in live weight and an estimated 8% (274 g) increase in feed consumption due to impaired Feed Conversion Ratio. The mortality rate within the modeled scenarios was calibrated at 8%. These findings suggest a substantial potential economic burden on the poultry industry. The analysis indicates that the majority of financial losses may stem from subclinical performance declines—specifically, impaired Feed Conversion Ratio and reduced growth—rather than acute mortality. These projections emphasize the urgent need for enhanced preventative strategies and subclinical monitoring to mitigate the estimated economic impact in Türkiye.

## Simple Summary

Coccidiosis is a parasitic disease that poses a major threat to poultry production and imposes significant financial challenges. This study provides an exploratory analysis of the economic dimensions of coccidiosis in the Turkish broiler industry, utilizing stochastic model-based projections informed by Monte Carlo simulation, data from 117 enterprises, and expert-elicited parameters. Probabilistic projections suggest that total national financial losses in Türkiye could reach an estimated mean of $15.1 million in the most likely scenario, with a stochastic range extending up to $46.3 million under pessimistic conditions. The results indicate that the majority of these financial im-pacts—approximately 62%—stem not from bird deaths, but from ‘hidden’ subclinical effects like reduced growth and poor feed efficiency. The model estimates an average financial burden of approximately $0.41 per animal. These exploratory findings highlight that prevention is not only about reducing mortality but also protecting the efficiency and economic health of production. Given the model-based nature of this study, these figures should be interpreted as strategic baselines rather than definitive values. Improved monitoring and preventative strategies are essential for the sustainability of the Turkish sector.

## 1. Introduction

The global human population is projected to reach 9 billion by 2050, rendering sustainable and secure protein production a primary objective of international food policies [1]. Poultry production has emerged as a critical pillar in addressing global protein demands [2,3]. However, intensive production models within broiler enterprises have exacerbated stress factors and increased disease prevalence. These challenges are primarily driven by high-density housing and the physiological demands of intensive production environments [4]. Consequently, any pathological outbreak that compromises poultry health not only disrupts production efficiency but also poses a significant threat to the integrity of the international food supply chain [5].

Coccidiosis is a pervasive parasitic disease in the poultry industry, characterized by a complex spectrum of intestinal damage ranging from subclinical malabsorption to severe hemorrhagic enteritis [4,6]. According to current taxonomic classifications, ten *Eimeria* species are recognized to infect broiler chickens—namely *E. tenella*, *E. necatrix*, *E. maxima*, *E. brunetti*, *E. acervulina*, *E. mitis*, *E. praecox*, *E. lata*, *E. nagambie*, and *E. zaria* [7]. The pathogenic impact of these species is highly variable; while *E*. *tenella* and *E*. *necatrix* are traditionally associated with high mortality and severe bloody lesions; other species, such as *E. acervulina* and *E. maxima*, induce significant malabsorptive damage, which is a primary driver of subclinical economic impacts. Recent research emphasizes that even without overt clinical signs, the impairment of intestinal integrity and the metabolic costs of the immune response lead to marked reductions in weight gain and feed efficiency [8,9,10].

The parasite completes its life cycle within the host over a period of 4 to 7 days. Transmission occurs via the fecal-oral route through the shedding of oocysts into the environment. These oocysts exhibit extraordinary environmental resilience, maintaining their infectivity in the litter or surrounding environment for extended periods despite fluctuating conditions [11]. Healthy birds become infected by ingesting sporulated oocysts from contaminated surfaces. Various environmental and management factors—including equipment hygiene, litter quality, humidity, multi-age housing, and nutritional status—play a critical role in the pathogenesis and spread of the infection.

The economic ramifications of the disease are directly correlated with its epidemiological prevalence. Coccidiosis remains one of the most significant financial burdens in the global poultry industry, with recent estimates exceeding US$14 billion annually [8]. This substantial economic burden has been further exacerbated by recent global crises, such as the COVID-19 pandemic and regional conflicts, which have notably inflated the prices of essential inputs, particularly feed and energy. Consequently, the economic impact of poor Feed Conversion Ratios (FCR) has become more severe, as the monetary value of feed loss has surged in direct proportion to rising commodity prices. Furthermore, disruptions in veterinary supply chains have hindered effective disease control, further deepening the overall economic damage [10].

In Türkiye, local studies substantiate these findings. For instance, research involving 622,000 broilers identified significant expenditures for coccidiosis control [12]. These figures underscore the financial pressure exerted by both direct expenditures (prevention and treatment) and indirect losses (performance degradation).

In the absence of such a national database, the structured Delphi consensus remains a valuable methodological alternative to bridge this information gap. While not a substitute for direct empirical measurement, the methodology is specifically designed to generate systematic projections in fields characterized by high uncertainty. The Delphi method is established in the scientific literature as a disciplined research instrument for aggregating expert opinions [13,14,15]. Its adaptation to health sciences has facilitated its transition to a reliable tool for aggregating ‘collective intelligence’ when direct data are constrained [16,17,18]. This technique has been successfully applied in the fields of veterinary medicine and animal health economics to prioritize zoonotic diseases, biosecurity measures, and the ranking of risk factors for various livestock pathologies globally [19,20,21]. Therefore, this study aims to provide an exploratory estimate of the economic impact of coccidiosis within Turkish broiler enterprises by integrating expert-elicited epidemiological parameters with localized financial data within a stochastic modeling framework informed by Monte Carlo simulation.

## 2. Materials and Methods

### 2.1. Data Collection and Methodological Framework

The methodology for estimating the economic dimensions of coccidiosis in this study was adapted from the framework established by Williams (1999) [22]. Primary data were collected during the 2024 production cycle through a multi-stage approach. Initially, operational data were obtained from commercial broiler enterprises across the Mediterranean, Marmara, and Central Anatolia regions, which represent the primary hubs of poultry production in Türkiye. To supplement these records and provide a robust estimation for parameters where direct field metrics were unavailable (such as subclinical weight loss and indirect performance impairments), expert opinion forms were distributed to a specialized cohort. This cohort included 24 experts, including veterinary specialists, integration-affiliated veterinarians, poultry health specialists, and industry representatives. The selection criteria for the panel required a minimum of 8 years of professional experience in the poultry sector, with participants’ experience levels ranging from 8 to 20 years. To ensure professional and geographical diversity, the panel was composed of specialists representing both academic institutions and private sector integrations across the three aforementioned production hubs. This panel was intentionally composed of specialists with extensive field experience in commercial operations to ensure that the model reflects the current practical realities of the industry. Veterinarians within large-scale integrations were prioritized as they possess the most granular, real-time data on subclinical performance and field challenges.

Economic impacts were subsequently quantified using the Delphi method based on these expert evaluations, serving as a strategic frame of reference for the national impact. The Delphi method was employed to achieve a consensus through a structured, iterative process of anonymous surveys conducted in two sequential rounds [23]. A three-week interval was maintained between rounds to allow for participants’ evaluation. Statistical consensus was quantitatively verified using the Coefficient of Variation (CV). While the first round showed higher variability (CV: 0.20–0.44), the second round resulted in CV values between 0.05 and 0.16, consistently remaining below the 0.20 threshold. This confirms a high degree of stability and consensus among the panel members, ensuring the reliability of the expert-derived inputs. Regarding ethical considerations, the Ethics Committee of Kırıkkale University reviewed the study protocol and officially waived the requirement for ethical approval, as the research did not involve experimental procedures on animals or sensitive personal data.

To account for the inherent uncertainties in expert-derived parameters and field data, the economic dimensions were estimated using a stochastic modeling framework. A Monte Carlo simulation consisting of 1000 iterations was performed by defining probability distributions (Triangular distribution: minimum, most likely, and maximum) for key variables such as prevalence, mortality, and weight loss. This simulation-based approach allowed for the generation of three distinct economic scenarios—optimistic, probable, and pessimistic—thereby providing a more robust risk assessment than a purely deterministic calculation.

### 2.2. Selection of the Research Area and Data Acquisition

This study was conducted in the Mediterranean, Marmara, and Central Anatolia regions, which represent the primary broiler production hubs in Türkiye. According to official reports from the Ministry of Agriculture and Forestry (MoAF) [24], these three regions collectively account for approximately 63% of the total broiler production in the country. Although the Aegean Region also maintains a high production capacity, primary operational data from enterprises were not directly sampled in this region due to logistical constraints and restricted data access from certain regional integrations. However, to maintain a national representative framework, the Delphi panel included experts with extensive professional experience and oversight in the Aegean Region, thereby ensuring that the parameter estimations reflect the broader national dynamics. A purposive sampling strategy was employed to recruit experts and acquire data from the key production regions that represent the majority of the national output. To minimize potential sectoral bias and ensure objectivity, the Delphi panel was strategically composed of a balanced mix of company-affiliated veterinarians, independent poultry health specialists, and academic experts with broad experience across the entire industry

The data acquisition process was executed in two primary stages during the first half of 2024. In the first phase, primary data were compiled from 117 broiler enterprises affiliated with three major poultry integrations. These enterprises were located in the Mediterranean, Marmara, and Central Anatolia regions, which provide a representative profile of the intensive broiler production sector in Türkiye. Additionally, direct on-site assessments were conducted at 14 representative farms to validate management practices and field conditions. These sources provided concrete data on epidemiological prevalence, mortality rates, and actual expenditures for treatments and prevention.

However, to ensure a comprehensive economic model, four specific parameters that are not typically isolated or recorded in commercial digital logs were determined through a structured Delphi consensus: live weight loss (g), feed consumption increase (g), treatment success rate (%), and duration of treatment (days). While the 117-farm dataset provided the financial and prevalence backbone of the study, the Delphi method was utilized to isolate the specific biological deviations caused solely by subclinical coccidiosis—data points that centralized databases currently lack the granularity to track. The absence of direct enterprise data from the Aegean region was strategically mitigated by the Delphi panel’s composition, which included experts with national oversight and specific field experience in Aegean production systems. This allowed the hybrid estimation model to bridge existing data gaps by integrating regionally focused empirical field records with expert-informed, national-scale stochastic simulations.

### 2.3. Delphi Method Application

#### 2.3.1. Methodological Justification and International Context

In the fields of veterinary medicine and animal health economics, the Delphi method is recognized as a fundamental methodology, particularly in managing complex epidemiological processes where objective and direct data are scarce. In the international literature, this technique has been successfully applied to the prioritization of zoonotic diseases in various geographies, such as Switzerland [19] and Australia [25], the classification of infectious diseases in cattle regarding biosecurity measures in Belgium [20], and the ranking of risk factors for subclinical mastitis [21]. In the context of Türkiye, Delphi expert surveys have constituted a critical data source for analyzing the financial impacts of Brucellosis [26] and Foot-and-Mouth Disease [27], as well as for cost–benefit analyses of control strategies. In this study, the method was specifically utilized to quantify parameters that are not typically captured by routine digital farm records, ensuring that the economic model remains grounded in the practical field experience of seasoned professionals while adhering to the statistical rigor of the iterative feedback process

The application of the methodology extends beyond disease management to multidisciplinary areas, including the determination of welfare indicators for shelter dogs [28], exploration of industry perceptions regarding equine welfare [29], and the development of competency-based veterinary education programs [30]. Furthermore, its use in critical medical processes, such as developing core outcome sets for clinical trials [31] and defining clinical management pathways for pediatric inflammatory syndromes [32], has reinforced its scientific credibility.

Although the Delphi method inherently carries a risk of subjective bias since it relies on expert opinions, this risk is minimized through participant anonymity, iterative feedback processes, and statistical analysis of group responses. Its primary advantage lies in eliminating the “halo effect” and dominance of specific individuals in committee-style meetings, thereby fostering a “collective intelligence” independent of hierarchy [17]. In cases like coccidiosis, where economic impacts are multidimensional (e.g., live weight loss, feed conversion ratio, medication costs, subclinical course) and field data cannot be fully tracked digitally via standard recording systems, Delphi serves as the most functional tool to transform isolated field experiences into meaningful economic data. This study was undertaken to bridge the data gap in the Turkish broiler sector by systematizing expert field knowledge where mainstream statistical methods remain limited. Adherence to reporting guidelines such as CREDES (Conducting and Reporting Delphi Studies) [33] further ensures the methodological integrity and scientific reliability of the research. To ensure the methodological integrity and scientific reliability of the research, the study adhered to the CREDES (Conducting and Reporting Delphi Studies) guidelines [33]. The potential for subjective bias was further mitigated through a two-round iterative process, where consensus was objectively verified using the Coefficient of Variation (CV). The final CV values (ranging from 0.05 to 0.16) consistently remained below the 0.20 threshold, confirming a high degree of stability in expert opinions. By adopting this hybrid approach, the study does not claim to present definitive national statistics, but rather provides a model-based framework that captures the practical field reality through the lenses of seasoned professionals.

#### 2.3.2. Expert Selection and Panel Composition

Specifically, a panel of 24 veterinary specialists was consulted to establish consensus on four specific parameters that are not explicitly isolated in commercial digital logs: post-infection live weight reduction (g), feed consumption increase (g), treatment success rate (%), and duration of treatment (days). While other epidemiological and economic data—such as disease prevalence, mortality rates, and expenditures associated with treatments—were derived directly from the integrated companies’ official records and field surveys, the Delphi process was utilized to provide a refined ‘strategic frame of reference’ for non-recorded biological variables. This methodological choice was necessitated by the current limitations of the national livestock data infrastructure, where high-granularity empirical monitoring for subclinical performance is currently unavailable. Consequently, the Delphi consensus serves as the primary scientific mechanism to bridge this information gap, transforming expert-elicited parameters into a validated sectoral estimation. While the panel features a significant representation from the private sector, this composition was intentional; in Türkiye’s current poultry infrastructure, veterinarians within large-scale integrations are the primary custodians of high-granularity field data. Their involvement was therefore prioritized to ensure that the model parameters are grounded in actual industrial performance rather than theoretical assumptions. To ensure the validity and reliability of the Delphi consensus, experts were selected based on pre-defined criteria aimed at maximizing technical knowledge and field experience. These qualification criteria are detailed in Table 1.

#### 2.3.3. Sectoral Distribution of Experts

The expert panel consisted of 24 specialists, selected to represent a comprehensive range of perspectives within the Turkish broiler industry and veterinary services. As detailed in Table 2, the majority of the panel (83.4%; *n* = 20) originated from the private sector, comprising enterprise-affiliated veterinarians, production managers, and industry consultants. While this distribution reflects the industry-led structure of Turkish poultry production, it is acknowledged that a high proportion of private-sector participants may prioritize production-oriented perspectives. To balance this, the remaining 16.6% (*n* = 4) provided insights from public and academic sectors to offer a broader regulatory and scientific context.

This composition aimed to provide a multidimensional evaluation of the expert-informed economic parameters. The consensus reached across these diverse sectoral backgrounds was objectively validated through the iterative Delphi process, ensuring that the synthesized projection remains representative of the broader industry reality. By transforming divergent expert opinions into a stabilized frame of reference, the process effectively addresses the data constraints currently present in the national livestock infrastructure.

#### 2.3.4. Data Collection and Iterative Delphi Rounds

Round 1: The selected experts were provided with structured ‘expert data elicitation forms’ designed to quantify the economic impact attributed to coccidiosis. These forms (Supplementary File S1) allowed for the systematic collection of data on prevalence, growth impact (live weight loss), FCR impairment, and prevention expenditures. All responses were kept strictly confidential to minimize social pressure and individual bias.

Feedback and Consensus Mechanism: Following the analysis of the first-round results, measures of central tendency and dispersion were identified. These findings were synthesized and presented back to the experts as a feedback report. To measure the level of agreement and confirm the stability of the results, a 5-point Likert scale was utilized. This iterative process is a validated scientific method widely used in veterinary economics to reach consensus and acquire reliable data where nationwide longitudinal field measurements are limited [26,27,34].

Round 2: During the second round, experts were provided with an anonymous statistical summary (median and interquartile range) of the initial responses. This stage allowed participants to reassess their input in light of the collective technical spectrum, a controlled feedback loop designed to reduce variance caused by individual misinterpretations or unique regional anomalies. The process was concluded after the second round as the responses reached a high level of stability, objectively verified by the narrowing of the Interquartile Range (IQR) and the stabilization of the Coefficient of Variation (CV) within the 0.05–0.16 range. This indicated that further rounds would yield diminishing returns in terms of precision, confirming that a ‘sectoral saturation’ point had been reached for the parameters under study.

Methodological Justification: The economic impact was quantified using a stochastic modeling framework based on Williams (1999) [22] parameters, integrated with Monte Carlo simulation. We acknowledge that the framework by Blake et al. (2020) [8] represents a significant global benchmark; however, the Williams (1999) [22] model was preferred for this study as it directly aligns with the specific performance variables (e.g., precise weight gain and FCR deviations) elicited from our Delphi panel. This choice allowed for a highly localized and precise analysis by integrating the distinct feed-to-meat price ratios and medication expenditures unique to the 2024 Turkish poultry market. This methodological approach was necessitated by the current limitations of the national livestock data infrastructure. While the Ministry of Agriculture and Forestry maintains rigorous records for notifiable infectious diseases, Türkiye currently lacks a centralized, high-granularity empirical monitoring system for subclinical performance at the farm level. Consequently, the structured Delphi consensus—objectively verified by the convergence of expert opinions—serves as the most robust scientific bridge to transform field-level biological losses into model-based national projections. By utilizing stochastic modeling to mitigate the inherent subjectivity of expert elicitation, this framework provides a validated and systematic alternative to mainstream statistical methods where longitudinal national databases are unavailable.

The iterative Delphi process, conducted over two rounds to quantify the economic impacts of coccidiosis, is summarized in Table 3.

The results demonstrate a notable degree of convergence among the experts regarding the selected economic parameters. A key indicator of this methodological convergence was the systematic reduction in the Interquartile Range (IQR) between the two Delphi rounds. The stabilization of median values within a narrower IQR and the achievement of stable Coefficient of Variation (CV) values provide a more consistent data baseline. This progression suggests that the feedback mechanism effectively facilitated a systematic alignment of initially divergent perspectives into an industry-informed framework, reducing individual variance while maintaining the anonymity that prevents social conformity bias. This convergence confirms that the panel reached a ‘sectoral saturation’ point, ensuring that the derived parameters are statistically robust for integration into the stochastic economic model.

Statistical consensus among the expert panel was quantitatively verified using the Coefficient of Variation (CV). While ordinal measures such as Kendall’s W are frequently utilized for ranking-based Delphi studies, the CV was prioritized over Kendall’s W in this research. This selection was based on the fact that our Delphi process focused on continuous quantitative variables (e.g., economic costs, weight loss parameters) rather than ordinal ranking scales. A CV value below 0.20 was established as the threshold for strong consensus. In the final round, the CV values for all key parameters stabilized between 0.05 and 0.16, confirming a high degree of agreement and stability in the expert estimates, thereby validating the reliability of the parameters used in the stochastic model.

#### 2.3.5. Scenario Development and Economic Loss Framework

Based on expert consensus and empirical field data, three scenarios were developed to evaluate the economic dimensions of coccidiosis in broiler production. These scenarios were specifically designed to account for the inherent uncertainties in biological parameters that are currently unavailable in centralized national databases. Economic impacts were categorized under three main headings: production performance declines, treatment expenditures, and prevention expenses.

The scenarios are defined as follows:

- Optimistic Scenario: Reflects conditions with minimal disease impact. Calculations utilize the lower bound of the Interquartile Range (IQR) from the expert consensus (Table 3). For example, a live weight loss of 120 g per broiler was applied.
- Pessimistic Scenario: Represents severe disease impacts with inadequate control measures. Calculations are based on the upper bound of the IQR from Table 3. For instance, a live weight loss of 180 g per broiler was assumed.
- Probable Scenario: Reflects current average field conditions using the median values derived from the expert consensus (e.g., a median live weight loss of 150 g per broiler).

In this context, biological and financial parameters were integrated into a stochastic modeling framework informed by Monte Carlo simulation. To address parameter uncertainty, a Triangular Distribution was defined for each key variable, utilizing the minimum (lower IQR), most likely (median), and maximum (upper IQR) values derived from the Delphi consensus. This simulation-based approach allowed for the generation of three distinct economic projections, providing a more robust risk assessment than a purely deterministic calculation.

### 2.4. Estimation of Coccidiosis Prevalence

The prevalence of coccidiosis was assessed across 117 commercial broiler enterprises located in the Mediterranean, Marmara, and Central Anatolia regions—the primary poultry production hubs in Türkiye. Data were gathered through systematic field visits and the retrospective analysis of institutional records conducted throughout 2024. These regions were purposively selected as they represent the highest concentration of commercial broiler production in Türkiye, constituting the nation’s primary industrial corridors. By focusing on these high-density hubs, the study captures the management dynamics and disease patterns prevalent in large-scale integrations, providing a representative baseline for the national poultry landscape.

#### 2.4.1. Clinical Surveillance

Broiler flocks were monitored for pathognomonic clinical signs of coccidiosis, including mucoid or bloody diarrhea, ruffled feathers, and growth retardation. These clinical assessments, conducted in conjunction with official enterprise-level diagnostic reports, were performed by specialized veterinarians to provide a qualitative clinical context for the subsequent economic projections. The proportion of suspected cases and the overall health status of each flock were systematically documented in health monitoring reports, serving as an empirical reference for the expert-informed parameters used in the model.

#### 2.4.2. Analysis of Enterprise Records

Diagnostic reports, therapeutic protocols, and mortality logs maintained by the enterprises’ veterinary staff were analyzed to extract key performance and health indicators. These institutional records, which include laboratory-confirmed findings and necropsy reports, provided quantitative data on diagnosed cases. This information was utilized to estimate robust prevalence rates, mortality rates, and treatment durations. These enterprise-specific data points served as a foundational empirical baseline to inform the stochastic economic model, reflecting the actual field conditions and biosecurity standards of the surveyed enterprises.

#### 2.4.3. Prevalence Estimation

Enterprise-level prevalence was defined as the percentage of enterprises with at least one confirmed coccidiosis case relative to the total number of enterprises surveyed within each specific region (Table 4). Similarly, bird-level prevalence was calculated as the ratio of the total number of affected animals to the total population under study. Each regional prevalence rate was determined independently to reflect the local disease burden; therefore, these percentages are specific to their respective regions and are not cumulative. While these prevalence rates are derived from direct empirical records of 117 enterprises, they provide a robust and representative baseline for the hybrid model. This ensures that the study’s primary focus—bridging the national data gap—is supported by high-fidelity field data, confirming that the derived figures reflect the industrial standards of Türkiye’s most intensive production zones during the study period.

As summarized in Table 4, the epidemiological data compiled from 117 commercial enterprises—covering the primary industrial clusters of Türkiye’s intensive broiler production—indicated a total study population of 1.66 million birds across the surveyed poultry houses. The regional distribution of these enterprises and their respective flock sizes highlights the significant scale of production in the Mediterranean, Marmara, and Central Anatolia hubs. This high-density sampling provides a robust and substantial empirical foundation for the subsequent stochastic economic estimation model.

### 2.5. Economic Loss Modeling and Mathematical Framework

The mathematical framework utilized to estimate the total economic dimension (Ctotal) attributed to coccidiosis in the Turkish broiler industry is summarized in Table 5.

This stochastic modeling framework, which incorporates both direct and indirect economic variables, was adapted from the framework established by Williams (1999) [22] integrated with Monte Carlo simulation to suit current local production data.

The stochastic model was executed using a Monte Carlo simulation framework developed in Microsoft Excel (Office 2021), utilizing a total of 1000 iterations to ensure the stability of the output distributions. Each iteration randomly sampled values from the predefined Triangular Distributions for key biological and financial variables. The Triangular Distribution was specifically selected as it is the most appropriate model for epidemiological contexts where empirical longitudinal data are scarce, allowing for the integration of expert-elicited values—specifically the minimum, most likely (median), and maximum—into a formal probabilistic structure. To maintain model parsimony and avoid over-parameterization, input variables were treated as independent parameters. This iterative process allowed for the transition from a deterministic point estimate to a robust probabilistic range, effectively capturing the cumulative variance of the economic impact.

This stochastic modeling framework, which incorporates both direct and indirect economic variables, was adapted from the framework established by Williams (1999) [22] and calibrated using Monte Carlo simulation to suit current local production data. The total economic dimension of the disease (C_total_) is projected as the sum of mortality-related losses, performance reductions (weight loss and FCR increase), and additional management or therapeutic expenditures. This framework provides a stochastic national estimation, utilizing probability distributions to reflect the potential economic scales based on the integration of field data and expert-elicited parameters.

While the study covers a significant portion of Türkiye’s broiler production, certain geographical and structural limitations are acknowledged. The exclusion of the Aegean Region represents a geographical and operational limitation, primarily stemming from restricted data access and a lack of institutional cooperation from regional integrations during the study period. Furthermore, as the empirical data were derived from 117 enterprises affiliated with three major poultry integrations, the results may reflect standardized management protocols and biosecurity similarities inherent to large-scale industrial systems. To mitigate these constraints and ensure a national perspective, the Delphi panel was intentionally designed to include experts with extensive field experience in the Aegean Region and diverse sectoral backgrounds. This hybrid approach allows the model to bridge geographical data gaps while acknowledging the operational specificities of the surveyed integrations.

## 3. Results

The technical and financial parameters and estimated values used in the calculation of the economic loss of coccidiosis in Türkiye are shown in Table 6.

The technical and economic parameters presented in Table 6 provide the foundational inputs to model the multifaceted negative impacts of coccidiosis on broiler production. Regarding the epidemiological baseline, the disease prevalence across the total study population (*n* = 1.66 million broilers) was identified as 13.3% (range: 10–22%) at the bird level. It is important to clarify that this figure represents the proportion of individual broilers affected by the disease, rather than a house-level estimation. This bird-level granularity was maintained throughout the model to ensure that performance-related losses (weight loss and FCR increase) were applied specifically to the infected population (n_inf_), providing a more precise and conservative economic projection.

These primary data were evaluated in two rounds with veterinarians via the Delphi process. This approach reflects a model-informed approximation of the broiler sector in Türkiye, providing a strategic frame of reference necessitated by the current limitations in national data infrastructure. To transition from the study population to a national scale, a linear extrapolation was employed. The unit loss per bird calculated from the study data was projected onto the total national broiler production for the year 2024. This extrapolation logic assumes that the production dynamics in the surveyed regions are representative of the intensive industrial broiler corridors of Türkiye.

According to the model-based projections summarized in Table 7, the economic dimensions attributable to coccidiosis were evaluated over three different scenarios. Under the probable scenario, the total economic impact for the study population (*n* = 1.66 million birds) was projected to be approximately $444,718. The average economic impact per animal was estimated at approximately $0.41, while the loss per clinically infected bird reached $2.06, based on the identified 13.3% bird-level prevalence.

By applying the national extrapolation framework, the total annual financial burden of coccidiosis on the Turkish broiler industry is estimated at approximately $15.1 million under the probable scenario (ranging from $10.8 million in the optimistic to $19.3 million in the pessimistic scenario). These findings suggest that coccidiosis poses a substantial financial burden in broiler production, with the majority of the impact stemming from “invisible” performance losses rather than direct clinical costs. Consistent with the exploratory nature of this work, these results represent scenario-based approximations necessitated by current gaps in the national livestock data infrastructure.

**Figure 1:**
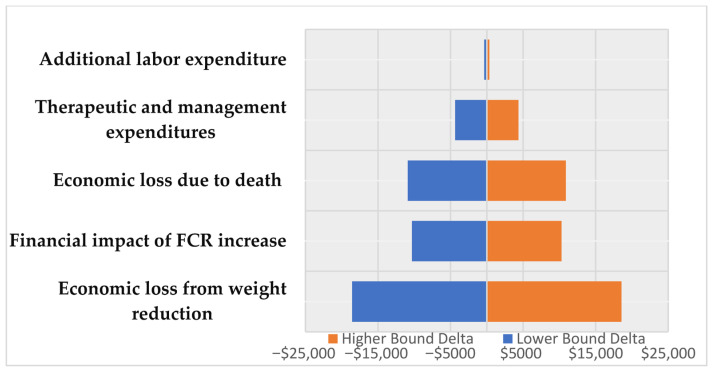
Tornado diagram showing the sensitivity of total economic loss to ±10% variation in key parameters (Baseline: $444,718).

To further evaluate the robustness of these projections, a formal deterministic sensitivity analysis was conducted by varying key input parameters by ±10%. The relative impact of each variable on the total economic loss is visualized in the Tornado chart (Figure 1). This analysis reveals that the model is most sensitive to fluctuations in live weight reduction and mortality rates, which are the primary drivers of the projected financial impact. In contrast, the model showed lower sensitivity to changes in additional labor and medication costs. This hierarchy confirms that biological performance parameters have a more decisive influence on the overall economic outcome than direct treatment expenses, highlighting the need for precision health management to mitigate subclinical effects.

The Tornado diagram illustrates the impact of a ±10% variation in key input parameters under the probable scenario. The central vertical axis represents the baseline value ($444,718), while the width of the bars indicates the relative sensitivity of the total loss to each variable.

These sensitivity results underscore the importance of precision in managing high-impact biological variables. While these projections should be interpreted as preliminary model-dependent estimations, they provide a strategic frame of reference for understanding the potential scale of economic impact. To address the challenges identified by the model, multifaceted protective measures are detailed for a 10,000-bird enterprise in Table 8, based on expert-informed parameters.

According to the expert-informed parameters presented in Table 8, the average cost associated with coccidiosis for a 10,000-head broiler enterprise was projected at approximately $654.4. Within the scope of hygiene and disinfection measures, the cost of regular cleaning and equipment disinfection was estimated at approximately $52. Interviews with breeders and expert consensus revealed that the combined use of coccidiostats and gut health supplements—such as probiotics, prebiotics, and mycotoxin binders—costs approximately $70.4. While these additives do not possess direct anticoccidial activity, they are utilized to support overall immunity, thereby indirectly mitigating secondary effects. Vaccination programs were generally less preferred by the panel due to their relatively high projected cost (about $532).

Although culling of infected broilers may be necessary in severe cases, field reports and expert consensus suggest that regular disinfection practices generally prevent infections from reaching critical levels; therefore, culling was not included in the model. However, an estimated average mortality rate of 8% was incorporated into the analysis, resulting in a projected loss of approximately 2.85 million broilers annually across Türkiye. Based on these parameters, the financial impact of this mortality is projected to represent an economic loss of approximately $3.6 million to the national economy, suggesting a potential financial burden of $142 for a 10,000-bird enterprise. To account for the inherent uncertainties in biological and economic variables, a Monte Carlo simulation was employed. The model was calibrated using expert-informed parameters to estimate the economic impact across three different scenarios: optimistic, probable, and pessimistic.

The damage caused by coccidiosis in the intestines of broilers typically results in financial losses by disrupting nutrient digestion and absorption. Based on expert consensus incorporated into the model, it was projected that infected animals have an average live weight loss of 150 g and an average increase in feed consumption of 274 g. These performance impairments were estimated to represent a potential economic impact of $6.137 million (due to live weight loss) and $3.408 million (due to increased feed consumption) on the national economy, respectively. For a 10,000-bird enterprise, the projected financial burden from live weight loss was approximately $242, while the expenditure associated with increased feed consumption was projected at $134. In line with the exploratory nature of this work, these figures represent a model-informed approximation designed to provide a strategic frame of reference where national empirical data are limited.

Extra labor time varies depending on disease severity and herd size. It was estimated that approximately 15 h of additional labor is required for monitoring, disinfection, and post-treatment care. This additional labor represents a projected financial impact of approximately $36 for a 10,000-bird enterprise and totals a projected $54,993 for Türkiye as a whole. Based on expert consensus, it was projected that a 5-day treatment was administered at an average expenditure of $0.009 per chick, resulting in a potential financial requirement of $57 for a 10,000-bird enterprise and an estimated aggregate economic impact of approximately $1.44 million on the national economy.

In this study, the total projected economic loss due to coccidiosis in Türkiye was estimated at $15.1 million in the probable scenario ($3.3 million optimistic to $46.3 million pessimistic). For a 10,000-bird enterprise, the total economic loss in the probable scenario was projected at $851. The analysis suggests that the average economic impact per animal was approximately $0.41 (range: $0.13–$0.83). These figures provide a strategic frame of reference for the Turkish poultry industry, acknowledging the exploratory and model-based nature of these estimations.

### National Economic Extrapolation Framework

The total economic impact (C_national_) was estimated by projecting the simulated study results onto the national production scale through a two-step linear scaling framework:

First, the cumulative simulated economic loss from the study population (C_total_) was divided by the total number of broilers in the sample (n_study_ = 1.66 million), yielding a unit loss per bird (u). This value serves as a weighted index capturing the integrated impact of mortality, subclinical performance drops (FCR and weight loss), and treatment costs. Second, this unit loss was multiplied by the total national broiler production of Türkiye (N_national_) for the year 2024, formulated as:

$$C_{national}=\;\left(\frac{C_{total}}{n_{study}}\right)\times \;N_{national}$$

To address the inherent uncertainties of field-derived data, the national projection is interpreted through a probabilistic framework rather than as a static figure. The reported national economic burden of 15.1 million USD represents the median output derived from a 10,000-iteration Monte Carlo simulation. This approach incorporates the variance in biological parameters and market prices across iterations, providing a more robust estimation than linear point calculations.

The model assumes that the biological and economic stressors observed in the 117 high-density production hubs—which represent the core of the country’s industrial capacity—are representative of the broader Turkish broiler sector. By integrating these field-based unit losses with national production statistics, the study provides a defensible strategic estimation of the disease’s total economic footprint in Türkiye.

## 4. Discussion

In this study, based on data obtained from 117 enterprises, the average prevalence of coccidiosis was projected at 13.3% across a total population of 1.66 million birds. This figure falls within the range of several regional studies conducted in Türkiye, which have reported prevalence rates between 7% and 57% [12,37,38]. The variations observed between these findings and our exploratory estimation may be attributed to differences in regional biosecurity practices, infection intensity, or the specific reporting protocols utilized by the enterprises. These differences highlight the model-dependent nature of such prevalence estimations in the absence of a centralized national surveillance system.

Global studies indicate that the prevalence of coccidiosis varies significantly based on regional conditions, hygiene standards, biosecurity measures, and climatic factors. Blake et al. (2020) [8] reported that *Eimeria* species are more prevalent in hot and humid climates, with prevalence rates reaching 50–80% in certain regions. Other studies have documented prevalence rates of 61.3% in Tanzania [39], 42.2% in Ethiopia [40], 52.9% in Nigeria [41], and 88.24% in North India [42]. The lower prevalence estimated in our exploratory analysis (13.3%) compared to these high global figures may reflect differences in regional poultry management and climate, while also highlighting the model-dependent nature of our projection compared to empirical site-specific surveys

The mortality rate in coccidiosis appears to vary depending on several factors, such as infection severity, the virulence of the *Eimeria* species involved, management conditions, and the immunological status of the birds. Literature reports suggest that mortality rates due to coccidiosis typically range from 1% to 15%; while this rate may be maintained within the 1–5% range in well-managed enterprises, it has been documented to rise to 10–15% in cases of severe infection [8,22,43]. A regional study conducted across Türkiye also indicates that coccidiosis mortality varies between 1% and 10% [38]. Our model-based assumption of an 8% average mortality rate aligns with these reported ranges, reflecting a conservative yet representative figure for the exploratory analysis of national impact.

As a critical input for the economic modeling of this study, and considering the wide range in the relevant literature and expert consensus, an average mortality rate of 8% was established as a representative base value. In the analysis, the projected financial losses due to coccidiosis mortality were calculated at approximately $3.6 million across the national economy and $142 for a 10,000-bird enterprise, representing approximately 16.6% of the total estimated economic impact. This finding is largely consistent with Blake et al. (2020) [8], who reported that mortality-related losses typically account for 10–15% of the total global economic impact. The slight variance in our exploratory model likely reflects regional specificities in the Turkish poultry sector and the expert-informed parameters utilized in the absence of national empirical data.

In the analysis, the average financial burden per bird due to coccidiosis was projected at $0.41 (range: $0.13–$0.83). This projection is broadly comparable to findings by Kinung’hi (2004) [39] and Lobago (2005) [43], who reported economic impacts per bird between $0.5 and $2. In contrast, a study in Sweden estimated the economic dimension at €0.023 per kg of live weight [44], highlighting how regional production costs and disease intensity can influence economic outcomes. Since field-derived empirical data on weight loss are often limited, expert elicitation was utilized to address these data gaps. In this context, the average live weight loss was estimated at 5% (range: 2–10%). This exploratory estimation is relatively consistent with Williams (1999) [22], who reported a 2.15% reduction, with the variance likely attributable to the model-dependent parameters and the specific infection pressures considered in our study.

*Eimeria* species alter intestinal functions and reduce absorption capacity, leading to significant economic impacts by impairing production performance [9]. The resulting infection causes deficiencies in nutrient absorption and decreases growth rates [45]. Coccidiosis induces live weight loss and negatively affects the feed conversion ratio (FCR) as birds cannot efficiently utilize nutrients. Since feed intake typically decreases during infection, impaired growth results from both physiological changes and reduced consumption—critical factors that increase the overall economic burden [8,22]. In this study, an average live weight loss of 150 g was incorporated into the model based on expert elicitation and qualitative assessments provided by the enterprises. While this projected value is higher than some findings reported by Williams (1999) [22] and Blake et al. (2020) [8], such variance is likely attributable to regional management practices, infection severity, and the specific exploratory parameters used to reflect the local conditions in Türkiye.

In this study, the projected financial impact of live weight loss and increased feed consumption on the national economy was estimated at approximately $6.137 million and $3.408 million, respectively. These exploratory findings appear higher than results reported by Williams (1999) [22] and Waldenstedt [44]. Such discrepancies may be attributed to evolving management practices, as well as regional economic parameters such as fluctuations in average bird selling prices and feed expenditures. In particular, the geographical variability of feed pricing and market dynamics is considered a significant determining factor when projecting the economic dimensions of coccidiosis within the specific context of the Turkish poultry sector.

In this study, the financial loss resulting from impaired feed conversion efficiency was estimated to account for approximately 22% of the total economic impact. This figure reflects the additional feed required to reach target weights due to the disease’s impact on nutrient absorption, rather than an increase in daily appetite. This projection is remarkably consistent with findings from a study conducted in India [36], which reported a similar proportion of 22.7%. The alignment between our exploratory model and existing literature further suggests that impaired feed efficiency remains a significant and consistent economic dimension of the disease across different poultry production contexts.

The development and effects of coccidiosis are influenced by numerous factors and lead to multifaceted challenges, including impaired feed conversion efficiency, live weight losses, and increased mortality rates, as well as declines in flock uniformity, disease resistance, meat quality, and skin pigmentation [37,46,47]. These effects significantly threaten both animal welfare and the economic sustainability of poultry enterprises. In this study, the total economic impact for a 10,000-bird enterprise was projected at $851, while the aggregate economic loss across Türkiye was estimated at approximately $15.1 million within the probable scenario. Reflecting this substantial impact on a global scale, Blake et al. (2020) [8] stated that the total economic burden of coccidiosis worldwide exceeds $14 billion. Our exploratory analysis suggests that Türkiye’s share in this global burden is a critical strategic consideration for national biosecurity and industry sustainability.

Furthermore, Blake et al. (2020) [8] emphasized that the majority of these global losses are driven by feed conversion inefficiency, mortality, and decreased production performance. The same study reported that the total economic impact in different countries varies significantly, ranging from approximately $16 million in New Zealand to $1.175 billion in the USA. In parallel, a study conducted in the UK estimated the aggregate economic burden of the disease at approximately £38.6 million; it was reported that roughly 80% of these losses were due to impacts on mortality, weight gain, and feed conversion, while approximately 17.5% were attributed to chemoprophylaxis and treatment expenditures [22]. These diverse international findings underscore the model-dependent nature of economic estimations and the influence of national production scales on the final projected values.

A variety of methods are employed within disease control policies, ranging from disinfection and hygiene protocols to vaccination and the use of natural anticoccidials. Studies such as Shivaramaiah (2014) [48] and Blake et al. (2020) [8] suggest that combining vaccines with anticoccidial drugs can provide effective control; however, they also note that rising anticoccidial resistance and associated costs may challenge the long-term sustainability of these strategies. Consequently, the potential importance of biosecurity measures is emphasized, suggesting that low-budget hygiene practices could substantially mitigate financial losses. These findings highlight that infection control is not merely a clinical challenge but a strategic economic balance within the enterprise’s management framework.

In this study, the expenditure for regular cleaning and disinfection was projected at approximately $0.0052 per bird. Additionally, the estimated investment in probiotics, prebiotics, mycotoxin binders, antioxidant supplements, and coccidiostats was calculated as $0.0070, while medication and treatment expenditures amounted to approximately $0.0056. Broadly aligning with these projections, Çiçek (2016) [12] reported expenditures for coccidiostat control at approximately $0.0074 and therapeutic requirements at $0.0043 per bird. The minor variances between these studies likely reflect fluctuations in input prices and the exploratory nature of the parameters used to model current national production costs.

While vaccination and disinfection expenditures were not evaluated in Çiçek’s study [12], the present research suggests that vaccination programs are not commonly adopted by many enterprises due to their perceived substantial financial burden. In our analysis, the average economic requirement for a vaccination program in an enterprise with a capacity of 10,000 birds was projected at $532. This finding indicates that cost remains a critical factor potentially affecting the economic sustainability of vaccination practices within the current market framework.

According to the calculations, the total expenditure for hygiene, disinfection, and coccidiostat use within the national economy of Türkiye was projected at approximately $3.1 million. This value is notably lower than the £11.2 million estimated in the probable scenario reported for coccidiosis control expenditures in the United Kingdom [47]. The disparity between these exploratory findings is likely attributable to differences in regional economic conditions, production systems, biosecurity standards, and the varying financial burdens of inputs. These factors underscore that economic projections are highly context-dependent and should be interpreted within the framework of national production scales.

Due to current limitations in national livestock monitoring systems, particularly regarding high-granularity data on subclinical infections, expert-elicited models offer a pragmatic framework for sectoral economic analysis in Türkiye. The methodological approach of this study, which integrates enterprise records with expert consensus, is consistent with established research in the Turkish veterinary field. For instance, Can and Yalcin (2011) [26] and Şentürk and Yalcin (2005) [27] successfully utilized expert elicitation techniques to address data gaps in financial impact assessments of Foot-and-Mouth Disease and Brucellosis, respectively. As noted by Şahin (2001) [34], such techniques serve as a robust strategic frame of reference in data-constrained research environments. By adopting this exploratory estimation approach, the present study provides a validated projection of the economic burden of coccidiosis, offering a baseline for further research despite the inherent challenges in nationwide data collection.

Since coccidiosis is a disease that cannot be entirely eradicated in poultry enterprises, prevention strategies primarily focus on minimizing subclinical losses. One of the most significant impacts of the disease is the reduction in production yield and the impairment of the feed conversion ratio. In this study, subclinical losses were projected to account for approximately 62% of the total economic burden. This exploratory estimation aligns with findings by De Gussem (2007) [49], who reported this figure at 70%. The consistency between our model-based results and global literature suggests that subclinical infections represent a substantial, yet often invisible, economic challenge within the poultry industry.

Diaz et al. (2002) [50] reported that losses are more pronounced in poorly managed enterprises, suggesting that deficiencies in regional biosecurity and management variability exacerbate these impacts. In particular, the challenges in diagnosing subclinical cases often compel enterprises to prioritize the implementation of comprehensive prevention policies [49]. In this context, strategic interventions—including improved diagnosis, targeted treatment, and the development of host immunity—have the potential to optimize operational expenditures and substantially mitigate the overall economic dimensions of the disease impact [12]. Adopting such early intervention strategies may serve as a critical pathway for enhancing the economic sustainability of poultry production in data-limited environments.

The development of effective biosecurity and educational programs across Türkiye represents a pivotal strategy for potentially reducing economic losses at the national level. Furthermore, adopting chemical and ionophore rotation strategies may help mitigate parasite resistance. At the enterprise level, management strategies—such as optimizing bird density, improving acclimatization conditions, and separating age groups—are essential considerations. Effective disease control is largely contingent upon regular field monitoring, fecal analysis, and the implementation of early detection mechanisms. By prioritizing training and veterinary counseling for personnel alongside enhanced biosecurity, it is possible to minimize the economic impact of coccidiosis while simultaneously fostering improvements in animal welfare.

Furthermore, the climatic diversity of Türkiye likely plays a significant role in the epidemiology of coccidiosis. *Eimeria* oocysts require specific temperature and humidity levels for sporulation. Given that Türkiye encompasses various climatic zones—ranging from the humid subtropical climate of the Black Sea region to the semi-arid conditions of Central Anatolia—it is anticipated that regional variations exist in disease prevalence and the resulting financial burden. These geographic factors suggest that national economic projections should be interpreted as weighted averages, reflecting a mosaic of diverse regional environmental conditions.

These environmental factors must be considered when developing localized biosecurity and control strategies. Consequently, the findings of this study should be viewed as a representative baseline rather than a universal constant. Due to the reliance on expert consensus and field data from specific regions, the reproducibility of these economic dimensions in different timeframes or territories is subject to variability. Therefore, a cautious interpretation is advised, and these results should be viewed as preliminary projections that warrant future empirical studies to further validate these findings across a broader range of production conditions.

### 4.1. Comparative Analysis of Control Strategies: Beyond Conventional Methods

The subclinical economic burden identified in this study emphasizes the necessity for high-precision prevention. Traditional live vaccines, while effective, rely on the controlled cycling of oocysts, which may inherently cause mild intestinal damage and temporary performance drops [8,51]. As noted by Quiroz-Castañeda and Dantán-González (2015) [52], modern broiler housing with high stocking densities facilitates rapid parasite dispersal, making the reliance on oocyst recycling a “double-edged sword” for gut health.

In contrast, next-generation multivalent and recombinant vaccines offer targeted immune protection by utilizing specific immunogenic antigens without inducing intestinal lesions [53]. By preventing initial mucosal disruption, these advanced technologies could theoretically eliminate the weight loss and FCR deterioration that drive the majority of the $15.1 million national loss. However, field applicability in the Turkish sector is dictated by the cost-scalability balance. Our findings show that vaccination expenditure ($532 per 10,000 birds) is currently perceived as a substantial financial burden. While recombinant vaccines provide superior immunity and flock uniformity [45], their higher market price remains a barrier.

From a strategic management perspective, high-tech vaccine investments must be weighed against long-term Return on Investment (ROI). Considering that global coccidiosis losses reach $2.4 billion annually—driven largely by production inefficiencies [52]—the prevention of “invisible” subclinical losses could justify the initial capital outlay. Furthermore, unlike controlled challenge models that often fail to capture real-world stressors [54], our study utilizes field-derived data from 117 enterprises. This offers a more representative reflection of the actual economic impact, highlighting the need for scalable biosecurity frameworks that are practically viable for the industry.

### 4.2. Future Perspectives and Biological Insights

The impaired FCR and body weight gain identified as primary loss drivers are rooted in the pathogen’s disruption of the intestinal architecture. Liu et al. (2023) [55] provide compelling evidence on the efficacy of plant-derived tannins, which enhance growth performance and immune status (IgG and IgM) by modulating intestinal flora. Given that our model attributes approximately 22% of the total economic impact to feed conversion inefficiency, natural additives could serve as a vital strategy to offset these specific losses.

The mucosal damage induced by *Eimeria* species also triggers intense oxidative stress. Essential oils, such as *Litsea cubeba* (LCEO), offer a promising alternative to traditional chemoprophylaxis by upregulating antioxidant enzymes (SOD and GSH-Px) and suppressing pro-inflammatory cytokines [56]. Such phyto-biostatistical insights are particularly relevant for mitigating the damage leading to the subclinical drops that account for 62% of the total economic burden.

Furthermore, the economic impact is linked to nutrient malabsorption and disrupted lipid metabolism. As explored by Liu Q. et al. (2025) [57], dietary supplements can fundamentally alter metabolic pathways and growth outcomes. Understanding these shifts provides a scientific basis for targeted nutritional interventions to counteract parasite-induced weight loss.

The observed prevalence variations across 117 enterprises (7% to 57%) reflect the influence of management and environmental factors. Rearing conditions and intestinal regionality significantly shape gut metagenome and microbiome signatures [58], explaining how different environments in Türkiye influence microbial resilience against *Eimeria*. Finally, as demonstrated by Hu et al. (2024) [59] in an alternative infection model, pathogens can exert prolonged physiological stress long after clinical recovery. This parallel underscores that the economic burden of coccidiosis is predominantly driven by subclinical drops that often go unnoticed without rigorous monitoring. Incorporating these multidisciplinary insights is essential for developing biosecurity frameworks that protect both animal welfare and economic sustainability in the Turkish poultry sector.

### 4.3. Methodological Limitations

This study is subject to certain methodological limitations that warrant a cautious interpretation of the findings. Key biological and performance-related parameters were derived from expert consensus via the Delphi method rather than direct longitudinal measurements. While this approach is defensible in data-constrained environments, it introduces inherent subjectivity and potential cognitive biases, such as anchoring effects. The expert panel was assembled using a purposive sampling strategy to ensure high field expertise; however, it predominantly reflected private-sector perspectives (83.4%), and the potential for non-response bias from invited stakeholders who declined to participate cannot be entirely ruled out.

To address parameter uncertainty beyond a simple deterministic model, a stochastic Monte Carlo simulation was employed. This approach allowed for the estimation of economic impacts across three different scenarios—optimistic, probable, and pessimistic—providing a broader perspective on the financial burden. While defining precise distribution functions for a stochastic approach can be challenging with expert-derived data, the simulation was calibrated using range estimates (minimum, most likely, maximum) provided by the panel to enhance the robustness of the projections.

Additionally, while the Delphi process was conducted over two rounds, ordinal consensus measures such as Kendall’s W were not utilized due to the continuous quantitative nature of the parameters. Instead, consensus was rigorously quantified using the Coefficient of Variation (CV), ensuring that the agreement was statistically grounded for numerical data. There is also a current absence of nationwide empirical validation for these expert-derived values.

Despite these inherent limitations, the present study offers the first comprehensive exploratory projection of the economic dimensions of coccidiosis in Türkiye. By synthesizing expert elicitation with existing enterprise data and stochastic modeling, it bridges a critical information gap and provides a strategic baseline for both policymakers and industry stakeholders. Ultimately, this research serves not as a definitive conclusion, but as a foundational framework for future large-scale empirical surveillance and more granular economic impact assessments in the poultry sector.

The composition of the expert panel, with a significant majority (83.4%) representing the private sector, is acknowledged as a potential structural bias. This distribution may orient the findings toward production-centric perspectives. However, this composition was a deliberate strategic choice, as integration of veterinarians and field experts in Türkiye is the primary holder of granular, real-time epidemiological and financial data. To mitigate potential bias, academic and public sector representatives were included to provide a regulatory counter-balance. The high degree of consensus reached across these diverse professional backgrounds suggests that the findings represent a realistic synthesis of the industry’s economic challenges.

## 5. Conclusions

This study provides a comprehensive exploratory projection of the economic dimensions of coccidiosis in Türkiye’s broiler sector by synthesizing expert consensus with stochastic modeling. The findings indicate that the disease imposes a significant financial burden, with national losses projected at approximately $15.1 million in the probable scenario. A critical revelation of our model is that 62% of this impact stems from subclinical performance drops rather than clinical mortality, highlighting the “hidden” nature of the economic drain.

Our analysis underscores that moving beyond conventional control methods is no longer a choice but a necessity for the industry. While traditional live vaccines remain a staple, the transition toward next-generation recombinant and multivalent technologies—which target specific immunogenic antigens without inducing intestinal lesions—offers a superior mechanism of protection and an enhanced long-term Return on Investment (ROI). Furthermore, the integration of sustainable, non-pharmacological interventions, such as plant-derived tannins and essential oils, provides a vital management strategy to mitigate oxidative stress and improve feed conversion efficiency.

Ultimately, these findings advocate for a multidisciplinary strategic shift in national animal health policies. Integrating field-based economic projections with clinical insights is essential for developing robust, scalable biosecurity frameworks. While future research utilizing nationwide empirical surveillance is required to refine these estimates, this study serves as a foundational framework for policymakers to prioritize gut health optimization and high-precision prevention strategies, ensuring both animal welfare and the long-term economic sustainability of the Turkish poultry sector.

## Figures and Tables

**Table 1:** Expert Selection Criteria.

Criterion No	Criterion Title	Description
Criterion 1	Professional Qualifications and Expertise	Participants must hold advanced expertise or specialized training in relevant fields, such as infectious diseases, public health, preventive medicine, or broiler production systems.
Criterion 2	Minimum Professional Experience	A minimum of 5 years of active professional experience is required in areas including animal disease management, poultry epidemiology, or preventive medicine within the broiler industry.
Criterion 3	Scientific and Research Contribution	Priority was given to candidates with a record of national or international peer-reviewed publications on relevant topics or active participation in sectoral research projects.
Criterion 4	Regional Knowledge and Competence	Participants were required to possess comprehensive and current knowledge regarding production systems and disease management practices, backed by extensive field experience across all major production hubs, including but not limited to the Mediterranean, Marmara, and Central Anatolia regions.

**Table 2:** Professional backgrounds and sectoral distribution of the expert panel members.

Institution Type	Professional Profile (n)	Total Experts (n)
Private Sector	Vet. surgeons (8), Clinic owners (4), Production managers (3), Farm owners (3), Consultants (2)	20
Public Sector	Provincial Dir. specialists (2), Academic faculty (1), Research institutes (1)	4
Total		24

**Table 3:** Expert consensus on epidemiological and economic parameters for coccidiosis across two Delphi rounds.

Economic Parameters Requested from Experts	Unit	Median (R1)	IQR (R1)	Median (R2)	IQR (R2)
Reduction in Live Weight (g)	gram/per broiler	165	60–280	150	120–180
Relative Body Weight Reduction (%)	%	6.0	3.0–12.0	5.0	4.0–8.0
Therapeutic Efficacy Rate	%	0.91	0.80–100	0.94	0.90–100
Additional Feed Intake (g)	gram	245	180–350	274 g	220–330

R1 and R2: Refer to the First and Second Rounds of the Delphi survey, respectively. IQR (Interquartile Range): Represents the range between the 25th and 75th percentiles. A narrowing IQR from R1 to R2 indicates an increasing consensus among the experts.

**Table 4:** Number of broilers and prevalence, rate of the disease in the participating enterprises.

No	Research Area	Number of Houses	Number of Broilers	Enterprise-Level Prevalence (%)
1	Düzce	15	160,000	12
2	Bursa-İzmit-Adapazarı	25	300,000	22
3	Antalya	6	420,000	12
4	Ankara-Kırıkkale	38	450,000	10
5	Bilecik	15	150,000	15
6	Bolu	18	180,000	10
Total/Mean		117	1,660,000	13

Note: Regional prevalence rates represent the proportion of affected houses within each specific region independently and are not intended to sum to 100%.

**Table 5:** Formula for the calculation of economic loss due to coccidiosis.

Economic Parameter	Mathematical Formulation
Mortality Count (nmort)	Total broilers × Coccidiosis rate × Mortality rate
Infected Broiler Population (ninf)	Number of sick broilers−Number of dead broilers
Financial Loss from Mortality (Cmort)	nmort × Economic value of a 21-day-old broiler
Economic Loss from Weight Reduction (Cweight)	ninf × Average body weight reduction (kg) × Market price per kg
Economic Impact of Feed Inefficiency (CFCR)	ninf × ΔFCR × Unit feed price
Additional Labor Expenditure (Clabor)	ninf × Additional labor hours × Hourly wage
Therapeutic Expenditures (Ctreat)	ninf × Daily treatment expenditure per broiler × Duration
Total Economic Dimension (Ctotal)	∑(Cmort + Cweight + CFCR + Clabor + Ctreat)

FCR (Feed Conversion Ratio): The efficiency with which the birds convert feed mass into increased body mass, calculated as total feed intake divided by weight gain.

**Table 6:** Technical and economic parameters.

Technical and Financial Parameters	Professional Profile (n)	Total Experts (n)
Number of chickens in Türkiye	254,147,577	[35]
Live weight of 42-day-old broilers (kg/head)	2800 g (2450–3050 g)	Field-Derived Data
Total feed intake of 42-day-old broilers (kg/head)	4580 g (4280–4920 g)	Field-Derived Data
1 kg live weight price (kg/head)	$1.25 (1.22–1.35)	Field-Derived Data
Unit feed price ($/kg)	$0.38 (0.35–0.42)	Field-Derived Data
Coccidiosis prevalence	(13.3) (range 10–22%)	Field-Derived Data
Days when symptoms are observed	21-day (15–35)	[36]
Mortality rate (%)	8% (range 1–15%)	Field-Derived Data
21-day live weight (kg/head)	1.012 (0.950–1.100)	Field-Derived Data
21-day daily feed intake (kg/head)	1.155 (1.100–1.200)	Field-Derived Data
21-day-old chick value	$1.275	Field-Derived Data
Live weight loss	5% (range: 4–8%)	Expert consensus (Delphi) (Probable Scenario)
Live weight loss (g)	140 g (110–180)	Expert consensus (Delphi) (Probable Scenario)
Rate of increase in feed consumption	8% (range 4–8%)	[22]
Increase in extra feed consumption (g)	274 g (155–360)	Model Calculation
Extra labor (hours)	7 h (5–10)	Field-Derived Data
Extra labor expenditure ($/hour)	$2.40	Model Calculation
Treatment success rate	94% (90–100%)	Expert consensus (Delphi) (Probable Scenario)
Daily treatment and management expenditure per chick per day	$0.009 (0.006–0.011)	Model Calculation
Duration of treatment (days)	5 days (3–8)	Expert consensus (Delphi) (Probable Scenario)

Note: All economic values have been converted from Turkish Lira (TL) to US Dollars ($) based on the exchange rate of 1 USD = 34 TL active during the study period.

**Table 7:** Model-based projections of economic losses due to coccidiosis across different scenarios.

Economic Loss ($)	Probable Scenario	Optimistic Scenario	Pessimistic Scenario	Loss Per Enterprise	%
Economic loss due to death	108,870	79,842	143,410	4.30	23.2
Economic loss from weight reduction	185,573	136,152	244,451	7.32	39.6
Financial impact of FCR increase	103,051	75,573	135,748	4.05	22.0
Additional labor expenditure	3563	2612	4694	1.09	5.9
Therapeutic and management expenditures	43,661	32,034	57,557	1.72	9.3
Total Loss	444,718	326,113	585,860	18.48	100
Economic impact per infected bird	2.06	1.51	2.71	0.41	-

Note: All economic values have been converted from Turkish Lira (TL) to US Dollars ($) based on the exchange rate of 1 USD = 34 TL active during the study period.

**Table 8:** Economic dimension of protective measures for a 10,000-head broiler enterprise in one production season.

Expenditure Items	Estimated Expenditure ($)
Feed additives	22 kg × $3.2 = 70.4
Disinfection expenditure	8 L × $6.5 = 52.0
Vaccine expense	$532.0
Total expenditure	$654.4

## Data Availability

All data used in the analysis are provided and appear in the submitted article.

## Supplementary Materials

The following supporting information can be downloaded at: https://www.mdpi.com/article/10.3390/ani16071096/s1. Supplementary File S1: Expert Data Elicitation Survey Form; Table S1: Coccidiosis data of the integrated enterprises.

## Abbreviations

FCR	Feed Conversion Ratio
CFCR	Economic Impact of Feed Inefficiency
CV	Coefficient of Variation
CLABOR	Incremental Labor Cost
CMORT	Financial Loss from Mortality
CTREAT	Therapeutic Expenditures
CREDES	Conducting and Reporting Delphi Studies
NİNF	Infected Broiler Population
IQR	Interquartile Range
MoAF	Ministry of Agriculture and Forestry
NMORT	Mortality Count
TL	Turkish liras
TURKSTAT	Turkish Statistical Institute
USD	United States Dollar
