# Supplementary material for: A Delphi-Based Exploratory Estimation of the Economic Impact of Coccidiosis in Turkish Broiler Production
Source: Animals (Basel). 2026 Apr 2;16(7):1096. doi: 10.3390/ani16071096 (PMC13072150; doi:10.3390/ani16071096)
Supplement: Supplementary file 1 [file animals-16-01096-s001.zip › Supplemental File S1.pdf]

<u>Expert Data Elicitation Survey Form</u>

Section A: Professional Profile

1. Stakeholder Category:

   [ ] Private Sector (Integrations/Clinics) [ ] Public Sector (Ministry/Academic)

2. Primary Region of Expertise:
3. Scope of Supervision: Estimated total number of broilers managed/consulted per year: __________

Section B: Epidemiological & Performance Data (Reference Year: 2023) Please provide your professional estimates for Broiler flocks:

- Prevalence Rate (%): What is the estimated percentage of flocks affected? __________
- Outbreak Frequency: Average number of clinical outbreaks per year per enterprise: __________
- Growth Impact: Estimated average live weight loss due to infection (grams or %): __________
- FCR Impairment: Estimated average increase in Feed Conversion Ratio (points): __________
- Mortality Rate (%): Direct mortality attributed to coccidiosis outbreaks: __________

Section C: Economic & Control Parameters

- Treatment Strategy: Primary intervention used (e.g., amprolium, toltrazuril): __________
- Treatment Cost: Average expenditure for medication per bird ($): __________
- Prevention Cost: Average expenditure for vaccines/anticoccidials per bird ($): __________
- Control Efficacy: Estimated success rate of current prevention programs (%): _________

Note: This document represents the survey instrument for **Round 1 (Initial Elicitation)**. The consensus-building Likert scale was part of the subsequent Round 2 feedback phase and is not included in this initial baseline form
